# Informing the development of a scoring system for National Health Service Clinical Impact Awards; a Delphi process and simulated scoring exercise

**DOI:** 10.1177/20542704231217887

**Published:** 2024-01-14

**Authors:** Gary Abel, Rob Froud, Emma Pitchforth, Bethan Treadgold, Lucy Hocking, Jon Sussex, Marc Elliott, John Campbell

**Affiliations:** 1 University of Exeter Collaboration for Academic Primary Care (APEx), University of Exeter Medical School, Exeter, UK; 2 Clinvivo, Edenbridge, Kent, UK; 3 89908 RAND Europe, Cambridge, UK; 4 1312 RAND Corporation, Santa Monica, CA, USA

**Keywords:** professional conduct and regulation, medical careers, non-clinical, other medical management, medical management, health policy

## Abstract

**Objectives:**

To establish principles informing a new scoring system for the UK's Clinical Impact Awards and pilot a system based on those principles.

**Design:**

A three-round online Delphi process was used to generate consensus from experts on principles a scoring system should follow. We conducted a shadow scoring exercise of 20 anonymised, historic applications using a new scoring system incorporating those principles.

**Setting:**

Assessment of clinical excellence awards for senior doctors and dentists in England and Wales.

**Participants:**

The Delphi panel comprised 45 members including clinical excellence award assessors and representatives of professional bodies. The shadow scoring exercise was completed by 24 current clinical excellence award assessors.

**Main outcome measures:**

The Delphi panel rated the appropriateness of a series of items. In the shadow scoring exercise, a novel scoring system was used with each of five domains rated on a 0–10 scale.

**Results:**

Consensus was achieved around principles that could underpin a future scoring system; in particular, a 0–10 scale with the lowest point on the scale reﬂecting someone operating below the expectations of their job plan was agreed as appropriate. The shadow scoring exercise showed similar levels of reliability between the novel scoring system and that used historically, but with potentially better distinguishing performance at higher levels of performance.

**Conclusions:**

Clinical excellence awards represent substantial public spending and thus far the deployment of these funds has lacked a strong evidence base. We have developed a new scoring system in a robust manner which shows improvements over current arrangements.

## Introduction

A scheme to reward senior doctors who make an outstanding contribution to supporting the delivery of National Health Service goals in the UK has been in place since 1948. Iterations of the scheme led to the ‘Clinical Excellence’ award arrangements, in place between 2004 and 2022. The Advisory Committee on Clinical Excellence Awards of the UK government's Department of Health and Social Care advises health ministers on these awards.^1^ The scheme is designed to reward senior doctors who deliver above the standards expected of a consultant, academic general practitioner, or dentist fulfilling the requirements of their post. Until 2022, around 300 new national awards were made by the Advisory Committee on Clinical Excellence Awards each year in England, at a recent annual cost of around £125 million ($154 million).^2^ Four levels of national award existed: bronze, silver, gold and platinum; each level being associated with a financial reward ranging from between £36,192 ($44,537, bronze awards) to £77,320 ($95,253, platinum awards). Applicants for awards were assessed on evidence they provided regarding their contribution to delivering high-quality service, developing high-quality service, leading and managing high-quality service, research and innovation, and teaching and training. Evidence from applicants was independently scored by members of 15 regional subcommittees; approaches to scoring are informed by published research evidence.^3^

Between March and June 2021, the Advisory Committee on Clinical Excellence Awards undertook a national consultation on the potential revision of the national awards scheme, with the implementation of proposals subsequently taking place in Spring 2022. A revision was intended to address concerns regarding equity of access to and opportunity in securing these awards. Changes introduced involve revision, rebranding and renaming of the scheme, a new focus on ‘clinical impact’, doubling the number of awards and an overall reduced value of individual awards.^4^ The 2022 application round adopted the pre-existing four-point scoring scale (Box 1). However, a core part of a revised scheme is to have a scoring system that is robust, equitable, able to distinguish between levels of excellence and aligned with the new scheme's overall goals.

Box 1. Overview of current application and assessment arrangements.

Sixteen regional subcommittees of the Advisory Committee on Clinical Excellence Awards in England and Wales, comprising professional, employer and lay members, assess applications for national-level awards.

Applications are assessed following the submission of evidence relating to five domains of performance:

1. Delivering a high-quality service
2. Developing a high-quality service
3. Leadership and managing a high-quality service
4. Research and Innovation
5. Teaching and training

Evidence is submitted by applicants, employers provide sign-off to show applications are supported, and citations and rankings may be provided by national nominating bodies for new applications. Previously employer rankings and citations, and citations from third parties, were included. The scoring and rankings by employers have been removed from 2021 but employers are still required to provide validation that the information presented by applicants is correct.

Each domain is independently scored by multiple trained assessors, using a four-point scale:

0 – does not meet contractual requirements or insufficient information produced to make a judgement

2 – meets contractual requirements

6 – over and above contractual requirements

10 – excellent

Here we present the findings of research designed to inform the development of a revised scoring system that complements our previously reported qualitative research.^5^

## Methods

We aimed to establish a set of principles that could be used to inform a new scoring system by employing an online Delphi process.^6^ We used the resulting findings to develop a new scoring scheme, which we piloted in a shadow scoring exercise using anonymised applications from previous rounds of clinical excellence awards.

### Delphi

For the purposes of this process, we utilise the definition of Fink *et al.* that an expert ‘… should qualify for selection because they are representative of their profession, have power to implement the findings, or because they are not likely to be challenged as experts in the field’.^7^ We recruited 50 panellists from the existing Advisory Committee on Clinical Excellence Awards scoring committee members (all of whom were approached for an expression of interest by the Advisory Committee on Clinical Excellence Awards on our behalf), and representatives of national professional bodies (identified by us using contact details in the public domain). We targeted people from a range of ages, genders, and ethnicities and the Advisory Committee on Clinical Excellence Awards committee members makeup, including lay, professional and employer committee members and both chair and ordinary members.

In each round, panellists were presented with a number of items (Table 1) which they rated on a scale of appropriateness on a 9-point Likert scale (1=‘highly inappropriate’ to 9=‘highly appropriate’). Panellist's confidence in these ratings was solicited, but this was predominantly rated highly and so findings related to confidence are not presented. Panellists were invited to provide feedback on each item as well as (in Round One) to suggest alternative wording. Between rounds, panellists were provided with a summary of other panellists’ item ratings as well as a summary of comments, thus encouraging members to reflect on their judgements in subsequent rounds, shifting individual opinions into group consensus. Ratings and comments were made anonymously such that other panellists and researchers were unaware which panellists had contributed which ratings, thereby reducing the limitations imposed by group dynamics whereby a few dominate.^8,9^ After each round, items were rated as either appropriate (median rating of appropriateness 7 or higher), unsure, or not appropriate (3 or lower), and whether or not there was any disagreement using the RAND/University of California at Los Angeles appropriateness method.^10^ Disagreement was based on the inter-percentile range (the difference between the 30th and 70th percentile appropriateness ratings), and the inter-percentile range adjusted for symmetry (see Appendix A).^10^ We considered there to be evidence of disagreement when the inter-percentile range was more than the inter-percentile range adjusted for symmetry. A high-level summary of the material provided and of revisions made in each round is included in Appendix A with the full text of the online Delphi process available online.^11^

**Table 1.** Items rated in the online Delphi exercise along with the median ratings of appropriateness and whether there was disagreement among panellists in their rating.

	Round 1		Round 2		Round 3	
	Median rating	Disagreement	Median rating	Disagreement	Median rating	Disagreement
**Section one – Definition of excellence**						
Applicants could be benchmarked against all clinicians working in the UK, for example, the scale descriptors could be prefixed by ‘Compared to all clinicians working in the UK, this applicant…’	5	Yes				
Applicants could be benchmarked against all clinicians eligible for an award, for example, the scale descriptors could be preﬁxed by ‘Compared to all clinicians eligible for an award, this applicant…’			6	No		
Applicants could be benchmarked against all clinicians applying for clinical excellence awards, for example, the scale descriptors could be prefixed by ‘Compared to all clinicians applying for clinical excellence awards, this applicant…’	6	Yes				
Applicants could be benchmarked against all clinicians working in similar jobs/roles, for example, the scale descriptors could be prefixed by ‘Compared to all clinicians working in similar jobs/roles, this applicant…’	7	No	7	No		
Applicants could be benchmarked against all clinicians, eligible for an award, working in similar jobs/roles, for example, the scale descriptors could be preﬁxed by ‘Compared to all clinicians, eligible for an award, working in similar jobs/roles, this applicant…’			7	No		
Applicants could be benchmarked against all clinicians working in this speciality in similar jobs/roles, for example, the scale descriptors could be preﬁxed by ‘Compared to all clinicians working in this speciality in similar jobs/roles, this applicant…’			7	No		
Applicants could be benchmarked against all clinicians with similar levels of experience, for example, the scale descriptors could be prefixed by ‘Compared to all clinicians working with similar levels of experience, this applicant…’	4	No	4	No		
Applicants should be judged on their own merits and not be benchmarked against their peers.	4	No	3.5	No		
According to the Advisory Committee on Clinical Excellence Awards Guidance for Assessors 2021, ‘Clinical excellence’ is about providing high-quality services to the patient. It is also about improving the clinical outcomes for as many patients as possible by using resources efficiently and making services productive. Applicants need to show our assessors evidence of how they have made services more efficient and productive, and improved quality at the same time, as well as demonstrating their role as an enabler and leader of health provision, prevention and policy development and implementation. Further specific detail in respect of this definition is provided in the relevant guidance material covering the five current domains of performance. Please rate the appropriateness of the current definition of clinical excellence used by the Advisory Committee on Clinical Excellence Awards.	7	No				
The deﬁnition of clinical excellence used in the assessment of applications for clinical excellence awards made by part-time workers should be amended to reﬂect the part-time nature of their role.	7	Yes	5	Yes		
The deﬁnition of clinical excellence used in the assessment of applications for clinical excellence awards should be the same for all applicants, but the scoring of applications made by part-time workers should be amended to reﬂect the part-time nature of their role, with the ﬁnancial reward made on a pro-rata basis.			7	No		
The deﬁnition of clinical excellence used in the assessment of applications for clinical excellence awards should be the same for all applicants, but the scoring of applications made by part-time workers should be amended to reﬂect the part-time nature of their role, with the full ﬁnancial reward awarded to successful applicants.			4	Yes		
To strengthen equality, diversity and inclusion in the assessment of applications national nominating bodies, and specialist societies should publish their anonymised data, scoring methodology and justiﬁcation of their internal processes.	8	No				
To strengthen equality, diversity and inclusion in the assessment of applications, the Advisory Committee on Clinical Excellence Awards regional subcommittees should publish their anonymised data, scoring methodology and justiﬁcation of their internal processes.	8	No				
**Section two – Scale descriptors**						
For each domain, scoring scales could be described relative to expectations of the job description, for example, ‘The applicant: is working below expectations of the job description; meets expectations of the job description; somewhat exceeds expectations of the job description; clearly exceeds expectations of the job description: outstanding contribution, substantially exceeding expectations of job description’.	8	No				
For each domain, scoring scales could be described relative to the reach of the applicant's contribution to National Health Service care, for example, ‘the extent of the applicant's reach within a domain is predominantly: local, regional, national, or international’.	7	No				
For each domain, scoring scales could be described relative to the signiﬁcance of the applicant's contribution to National Health Service care, for example, ‘the signiﬁcance of the applicant's contribution within a domain is predominantly: not of substantial signiﬁcance, of some signiﬁcance, of substantial signiﬁcance, or highly signiﬁcant’.	7	No				
For each domain, scoring scales could be described relative to the impact of the applicant's contribution to National Health Service care, for example, ‘the impact of the applicant's contribution within a domain is predominantly: not of substantial impact, of some impact, of substantial impact, or highly impactful.	7	No				
For each domain, The description of scale points could be based on multiple aspects simultaneously, for example, ‘an applicant scoring at the highest point on the scale would be seen to be making an outstanding contribution, which is substantially exceeding the expectation of job description, highly impactful, highly signiﬁcant, and of international reach.’	7	No	5.5	No		
For each domain, The description of scale points could be based on multiple aspects simultaneously, for example, ‘an applicant scoring at the highest point on the scale would be seen to be making an outstanding contribution, which is substantially exceeding the expectation of job description, highly impactful, highly signiﬁcant, and of national or international reach.’			8	No		
The description of scale points could be based on statistical deﬁnitions, for example, ‘An applicant scoring at the highest point on the scale would be performing: in the top 1%.'	3	Yes	3	No		
The description of scale points could be based on statistical deﬁnitions, for example, ‘The performance of an applicant, in terms impact, signiﬁcance, and reach, relative to the expectations of their job description, at the highest point on the scale is judged to be: in the top 1%.'			4	No		
**Section three – Scoring**						
For each score given, an unevenly spaced small number of possible scores should be allowed, for example, the current scoring system of 0, 2, 6, or 10.	7	No	7	No		
For each score given, an evenly spaced small number of possible scores should be allowed, for example, 1, 2, 3, 4, or 5.	5	No	5	No		
For each score given, an evenly spaced larger number of possible scores should be allowed to permit more granularity, for example 0, 1, 2, 3, 4, 5, 6, 7, 8, 9, or 10.	5	Yes	5	No		
For each score given, an evenly spaced larger number of possible scores should be allowed to permit more granularity, for 0, 1, 2, 3, 4, 5, 6, 7, 8, 9 or 10 with clearly deﬁned anchor points for an unevenly spaced small number of points (e.g. 0, 2, 6 and 10).			6.5	No		
For each score given, an evenly spaced larger number of possible scores should be allowed to permit more granularity, for 0, 1, 2, 3, 4, 5, 6, 7, 8, 9 or 10 with clearly deﬁned scale descriptors covering a range of points.			7	No		
For each score given, an evenly spaced large number of possible scores should be allowed, to permit a high level of granularity, for example, a 0 to 100 scale.	2	No				
The lowest score on a scale should reﬂect someone operating below expectations.	4	Yes	4	Yes		
The lowest score on a scale should reﬂect someone operating at expectations	6	No	6	Yes		
For each score given, an evenly spaced larger number of possible scores should be allowed to permit more granularity, for example, 0, 1, 2, 3, 4, 5, 6, 7, 8, 9 or 10. Clearly, deﬁned scale descriptors covering a range of points should be provided with the lowest point on the scale reﬂecting someone operating below the expectations of their Job Plan.					7	Yes
For each score given, an evenly spaced larger number of possible scores should be allowed to permit more granularity, for 0, 1, 2, 3, 4, 5, 6, 7, 8, 9 or 10. Clearly, deﬁned scale descriptors covering a range of points should be provided with the lowest point on the scale reﬂecting someone operating at the expectations of their Job Plan.					6	No
Every applicant should provide evidence of excellence in all ﬁve domains, with scores for all ﬁve domains contributing equally to their ﬁnal score.	5	Yes	5	Yes	6	Yes
Every applicant should provide evidence of excellence in all ﬁve domains, with scores for their four highest-scoring domains contributing to their ﬁnal score.	6	No	6	No	7	No
Applicants may provide evidence of excellence in only four domains, with scores for these four domains contributing to their final score.	5	No	5	No	4	No
In a situation where applicant's ﬁnal scores were based on only four domains, these four domains must include the ‘service delivery and service development’ domain.	7	No				
**Section four – Alternative scoring approaches**						
Scorers will be asked to provide scores for individual domains as well as a single global score reﬂecting their overall impression. This single global score may deviate from the individual domain scores when felt to be appropriate. Total scores will be based on a combination of individual domain scores and the global score.			6	No		
Individual scorers should rank applications based on their overall impression of the application. Total scores for applicants would then be based on the average rank across individual scorers.			3	No		
Individual scorers should rank applications for each domain in the application. Total scores for applicants would then be based on the average rank across individual scorers and across domains.			3.5	No	4	Yes

### Shadow scoring exercise

To support the shadow scoring exercise, we developed a portfolio of training cases based on actual historical applications. Cases included both those that historically proved to be successful or unsuccessful and both new and renewal applications at various levels of awards. Cases were anonymised by removing all names of individuals and employers. Where details of research outputs were included author lists were replaced with the number of authors and the position of the applicant within the author list and as a lead author (first, corresponding or last author). Historically, applicants have been able to include citation text provided by employers, individuals, recognised national nominating organisations and specialist societies.^12^ Nominating bodies/specialist societies would also provide a ranking of the application relative to all supported applicants. Cases were based on 20 applications, where for 18 of which, two versions were created, one including all material, and a second with employer citation and ranking material removed (two applications did not contain citation material), that is, a total of 38 training cases. The historical scores given to each case, by each assessor, when the original application was made were made available to the research team.

Twenty-four experienced assessors of clinical excellence awards formed membership of a shadow scoring panel recruited for purposes of the research (recruited in the same way as Delphi panellists, including eight who were involved in both aspects of the study). Each member was sent 20 training cases and asked to score each of them using an online submission form representing a proposed novel scoring scheme developed following the Delphi exercise. Each of the 20 cases represented a different application, with each case being randomised to include a citation and ranking material or not on an assessor basis (i.e. some assessors saw an application with citations, while others saw it with citations removed and all assessors saw a mix of cases with and without citations). For the two cases where no citation material was available, all assessors were asked to score the same version. The order in which assessors were asked to score cases was randomised for each assessor to remove any order effects. We selected assessors to maximise diversity in terms of age, gender, ethnicity and committee role. In addition to scoring the applications, assessors provided feedback on the guidance, revised scoring and descriptors, using a structured set of questions. Further details of the scoring exercise are in Appendix B.

We employed multi-level regression models to examine the variance in scores given to applications that were either attributable to differences in the quality of the application itself, attributable to some assessors scoring systematically higher or lower on all applications, or unexplained/residual variance. We then considered only the variance attributable applications themselves and the residual variance and estimated what percentage of the sum of these two sources was attributable to the application. We did this separately for the scores from both this shadow scoring exercise and from original historic scores. The higher the percentage attributable to the application, the more reliable a scoring system is at distinguishing good and poor performance. Finally, we augmented models with an indicator of whether citation material was included or not, seeking to estimate the mean difference in scores reported when citation material and associated rankings were present. We provide further details of statistical analyses in Appendix C.

### Oversight

We established a three-member advisory group for the project that met with members of the research team in August 2021 and February 2022. The research team also worked closely with the Advisory Committee on Clinical Excellence Awards (meeting monthly senior representatives of the Advisory Committee on Clinical Excellence Awards) to access data to inform the development of training cases and in the recruitment of some participants. We also worked with a six-member PPIE group, throughout the research. Members, three women and three men, were from varied employment and professional backgrounds and a mix of working and retirement age.

## Results

### Delphi

Forty-five (90%) of those invited participated in Round 1 of the process. Of those, 10 were women (22%). The average age of participants was 57.18 years (standard deviation = 6.74). Twenty-eight (62%) identified ‘Health service provider (e.g. Hospital, general practice)’ as their place of primary employment; seven (16%) selected University; four (9%) selected public sector or government body (e.g. Department of Health, National Health Service England Clinical Commissioning Group, etc.); and six (13%) selected ‘Other’. Thirty-five (78%) participants described themselves as White, with the remainder describing themselves as Asian. Thirty-six (80%) participants had experience of being an Advisory Committee on Clinical Excellence Awards committee member and 12 (27%) participants represented professional bodies or representative groups (with three panellists declaring both roles). Of these, 40 panellists completed Round 2, and 39 completed all three rounds.

Further details of the Delphi process are provided in Supplemental Appendix D. Median appropriateness ratings and disagreement status for the items rated in each round are shown in Table 1. Based on these findings, we established a set of principles on which a future scoring system may be based (Box 2). Based on these six principles, we developed a new scoring system and guidance for assessors which was employed in the shadow scoring exercise.

Box 2. Six scoring principles emerging from the Delphi process.

**Figure 1.**
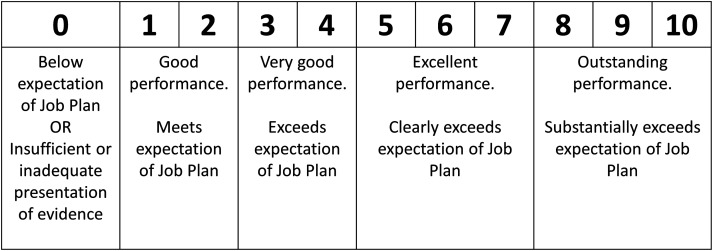
Example of application of scale descriptors for scale 0 to 10.

(i) The definition of clinical excellence as laid out in the Advisory Committee on Clinical Excellence Awards Guidance for Assessors 2021^13^ is appropriate, namely “Clinical excellence is about providing high-quality services to the patient. It is also about improving the clinical outcomes for as many patients as possible by using resources efficiently and making services more productive. Applicants need to show our assessors evidence of how they have made services more efﬁcient and productive, and improved quality at the same time, as well as demonstrating their role as an enabler and leader of health provision, prevention and policy development and implementation.” Further speciﬁc detail in respect of this deﬁnition is provided in the relevant guidance material covering the ﬁve current domains of performance.

(ii) The definition of clinical excellence used in the assessment of applications for clinical excellence awards should be the same for all applicants, but the scoring of applications submitted by part-time workers should be amended to reflect the part-time nature of their role, with the financial reward made on a pro-rata basis.

(iii) For each domain, the description of scale points could be based on multiple aspects simultaneously and these aspects would include their performance relative to the expectations of their job description, the reach of an applicant's contribution to National Health Service care, the significance of an applicant's contribution to National Health Service care, and the impact of an applicant's contribution to National Health Service care. For example, ‘an applicant scoring at the highest point on the scale would be seen to be making an outstanding contribution, which is substantially exceeding the expectation of job description, highly impactful, highly signiﬁcant, and of national or international reach.’

(iv) For each score given, an evenly spaced larger number of possible scores should be allowed to permit more granularity than exists in current scoring arrangements, for example, 0, 1, 2, 3, 4, 5, 6, 7, 8, 9 or 10 (compared with the four-point scale (0, 2, 6, and 10) in present arrangements). Clearly, deﬁned scale descriptors covering a range of points should be provided, with the lowest point on the scale reﬂecting someone operating below the expectations of their Job Plan (see Figure 1 for example).

(v) Every applicant should provide evidence of excellence in all ﬁve domains, with scores for their four highest scoring domains contributing to their ﬁnal score, but with the condition that the domains concerning service development and delivery were part of the scoring domains.

(vi) As part of the scoring process, applicants need to be benchmarked against their peers. However, there were three acceptable approaches to defining those peer groups: applicants could be benchmarked against all clinicians working in similar jobs/roles; all clinicians, eligible for an award, working in similar jobs/roles; all clinicians working in their speciality in similar jobs/roles.

### Shadow scoring exercise

**Figure 2.**
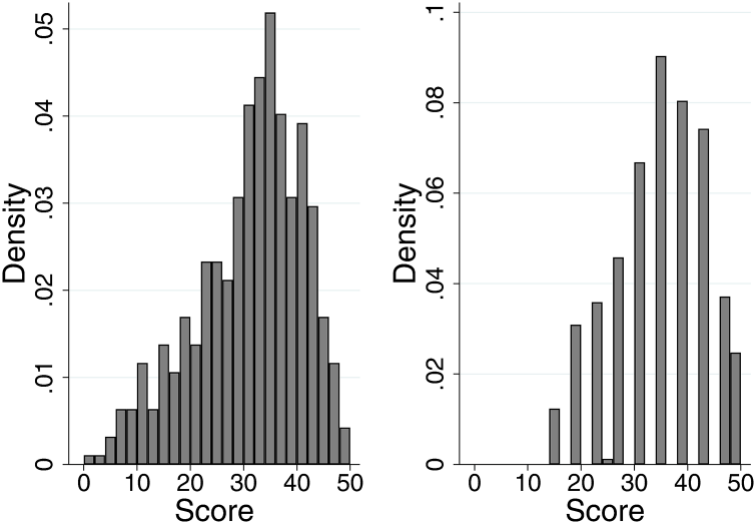
The distribution of scores assigned to the 20 applications by assessors in the shadow scoring exercise using the new scoring system (left, mean 30.6, range 1 to 50, interquartile range 24.5 to 38) and when originally considered for a clinical excellence award using the original scoring system (right, mean 33.9, range 14 to 50, interquartile range 26 to 42).

**Figure 3.**
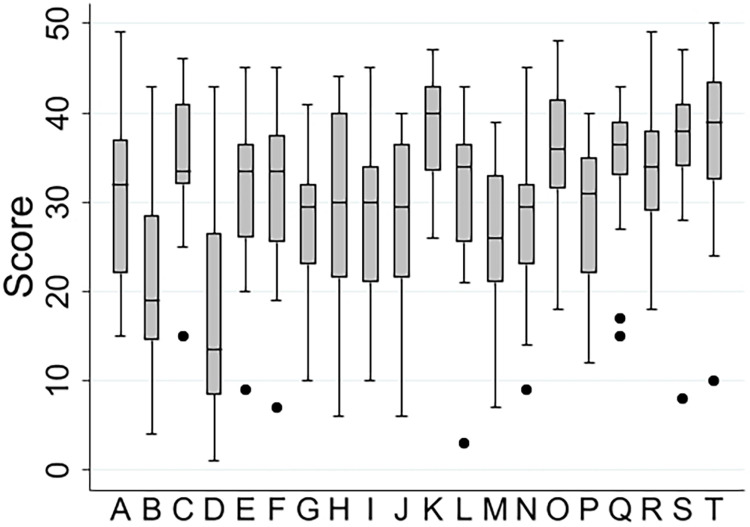
Box and Whisker plot showing the distribution of scores given to individual training cases by the 24 assessors.

The shadow scoring exercise yielded a total of 472 scores given by 24 assessors for 20 cases (eight scores were unusable due to missing case identifiers; Figure 2 left panel). The distribution of historic summary scores using the original scoring system shows that many possible values are not used (Figure 2 right panel), a pattern not exhibited with the new scoring system where assessors have utilised the fuller range of scores available. There was less of a ‘ceiling effect’ with the new scoring system than is seen in the historic scores. The results of the mixed effects models are shown in Table 2. The total variance is larger for the new scoring system reflecting a larger range of scores used. However, 48% of the variance in individual scores was attributed to assessors when using the new scoring system suggesting that there may be more hawkish/dovish scoring than with the current scoring system, which can be seen in the distribution of scores for individual applications (Figure 3). When the variance attributable to assessors is ignored, a slightly smaller percentage of variance attributable to the application in the new scoring system (46%) than the current system (51%) is evident, but the confidence intervals on these estimates overlap (new scoring system 95% CI 39.3% to 53.4%, current scoring system 95% CI 40.5% to 62.0%) implying that there is no statistically significant difference in the reliability of the scales used in this way to distinguish between good and poor performance. With the new scoring system, we found that when applications scored higher there was less variability in scores between assessors (correlation coefficient between variance and mean = −0.72, *p* < 0.001). This implies that discrimination might be better for higher-scoring applications where there is more agreement between assessors on clinical excellence. No such correlation was found for the historic scores. Finally, we observed a high degree of correlation (correlation coefficient = 0.77, *p* < 0.001) between the old and new scoring system scores.

**Table 2.** Variance components for application scores when using the new and current scoring systems.

	Considering all sources of variance				Considering variance attributable to the application and residual variance only			
	New scoring system		Current scoring system		New scoring system		Current scoring system	
	Variance	% of total	Variance	% of total	Variance	% of total	Variance	% of total
Assessor	49.5	48.3	30.8	35.5	–	–	–	–
Application	24.6	24.0	28.6	33.0	24.6	46.4	28.6	51.2
Error/residual	28.5	27.8	27.3	31.4	28.5	53.6	27.3	48.8
Total	102.6	100	86.7	100	53.1	100	55.9	100

When considering the effect of citations and associated ranking material on scores, we found no evidence that scores were any different when citations were included (mean difference 0.65-point increase, 95% CI −0.42 to 1.73, *p* = 0.234) compared with applications without citation material. Furthermore, there was no evidence from a random slope model that the effect of citations varied by case.

Assessors were generally positive about the new scoring system and the guidance provided (further details in Supplemental Appendix E). Just over half of the time, citation material (when present) was considered to be useful. The most commonly reported time spent coming to an assessment of an application was 10–20 min (45% of assessments), with just 2% taking over an hour.

## Discussion

Utilising a consensus-based approach to gain the views of experts, we identified six key principles which, we suggest, should be reflected in any revised approach to assessing and scoring applications for national clinical excellence awards. These comprise: (1) adopting the current definition of what constitutes clinical excellence as outlined in relevant documentation and guidance^13^; (2) ensuring that scoring of applications takes account of less than full-time working arrangements and making awards available on a pro-rata basis; (3) providing clear scale descriptors to ensure applications are scored relative to the expectations of the applicants' job plan and reflect the reach, significance and impact of an individual's contribution; (4) adoption of an extended scale to facilitate a more granular approach to the scoring of applications; (5) provision by applicants of evidence across five domains, but with only their top four highest scoring domains contributing to their final score, and with evidence of clinical service contribution being mandatory; and (6) benchmarking of final scores against those of peers working in similar jobs/roles.

A shadow scoring exercise based on an assessment of experimental applications by a constituted panel of experienced assessors identified potential benefits of a proposed new 10-point scoring scale. Observed benefits (compared with existing scoring arrangements) included: the utilisation of a wider range of scores, less evidence of a ceiling effect in scoring and a similar reliability of scores overall, while potentially better differentiation at higher levels of performance, where needed most. However, given the large variation between assessor scores and the relatively low variability attributable to applications concerns remain over the number of assessors needed to reliably score applications.^3^ The inclusion of citation material appeared to add little valuable information to inform scoring. Overall, assessors were positive in their views of the proposed new scoring arrangements.

### Strengths and limitations

We report findings from the most rigorous research programme yet commissioned to inform the development of the national clinical excellence awards scheme in the UK. The scheme is unique in terms of scope and the extent of the publicly funded financial awards made under the scheme. A robust and reliable approach to the assessment of applications is warranted. We adopted a multi-method approach to the research, utilised experienced assessors to support the research, and reported additional findings in the Supplemental Material.

The research was commissioned and undertaken at pace and benefited from advice from senior staff who were part of the UK body overseeing the potential revision of the national clinical excellence awards scheme. While they did not influence the research agenda or reporting of findings, they had an early sight of emerging results offering potential for preliminary findings to inform a revision of the scheme to focus on national impact.

Alternative, but more costly, approaches might have involved double-blind scoring of actual (rather than experimental) applications within the ‘live’ application and assessment process, but this would present financial, organisational and scientific challenges, which were not surmountable within the timescale available. Reflecting this compromise, we note that historical scores were based on complete applications including information removed for anonymisation, which may account for some differences between the two scoring systems.

While it is possible that assessors may have been involved in scoring an included application when it was originally made, the number of applications made each year across 15 subcommittees suggests this would be rare. Furthermore, given that cases were derived from 2009–2019 applications, the impact of recall is likely minimal.

### Policy implications

The UK National Clinical Excellence Award scheme is costly. It has been criticised as being elitist, and questions have been asked regarding the scheme's value for money.^14,15^ Specific concerns have been raised^16^ (and acknowledged by the Advisory Committee on Clinical Excellence Awards ^17^) regarding the accessibility of the scheme to all potential beneficiaries, mostly notably to women, to those working less than full time, and to senior doctors from ethnic minority backgrounds. The focus of this research was to inform the development of new scoring arrangements that are robust and equitable.

To date, a revised scoring system incorporating all the principles derived through this work has not been implemented. The revised and rebranded scheme implemented in 2022 by the UK Department of Health and Social Care focuses on clinical ‘impact’ rather than on clinical ‘excellence’, and the impact was indeed one of the key domains our research identified as being of key concern to our research participants. These important scheme changes were informed, at least in part, by the findings of a national consultation exercise which ran in parallel to this research.^18^ Other areas to consider as part of a revised scheme should include the reach and significance of an applicant's contribution – reflecting contributions beyond the immediate local geographical boundary of an individual's role, and the individual's contribution to influencing health and health care. Furthermore, a transition from the pre-existing, four-point, scoring scale to a more granular 0–10 scale has not yet been implemented. Given the similar levels of reliability found here between the two scoring systems that may be prudent. However, given that historic scores were provided by assessors who were well versed and trained in using that scoring system, and with some information having been removed for purposes of maintaining anonymity, there is potential for the reliability of scores using the proposed scoring system to be improved with training and use. A larger-scale prospective evaluation of the proposed scoring system, accompanied by full training in its use, is warranted.

## Supplemental Material

sj-docx-1-shr-10.1177_20542704231217887 - Supplemental material for Informing the development of a scoring system for National Health Service Clinical Impact Awards; a Delphi process and simulated scoring exercise Click here for additional data file. Supplemental material, sj-docx-1-shr-10.1177_20542704231217887 for Informing the development of a scoring system for National Health Service Clinical Impact Awards; a Delphi process and simulated scoring exercise by Gary Abel, Rob Froud, Emma Pitchforth, Bethan Treadgold, Lucy Hocking, Jon Sussex, Marc Elliott and John Campbell in JRSM Open

## Appendix A. Further details of the Delphi process

### Methods

In Round 1 (15 July 2021–29 July 2021), panellists were provided with a summary of academic literature pertaining to Clinical Excellence Awards, the findings of the INvestigating Clinical Excellence Awards (INCEA)project evidence review into performance-related financial incentive schemes, and a summary of the preliminary findings from the qualitative interviews conducted at that time. They were then asked to rate and comment on 25 items pertaining to the definition of excellence and potential scoring approaches. These items were based on discussions between the research team and informed by qualitative work. After completion of the round, the research team reviewed scores and free text comments and discussed which items would be taken forward to subsequent rounds.

In Round 2 (19 August 2021–2 September 2021) and Round 3 (23 September 2021–11 October 2021), panellists were provided with a written summary of the findings from the previous round. This included both a summary of the appropriate ratings of items and a summary of the free text comments including a selection of illustrative quotes. Items were not presented for rating again when the median was in either ‘appropriate’ or ‘inappropriate’ and there was no disagreement on the item and no comments that researchers judged required review by the panel. Some item wordings were altered following suggestions by panellists, and in some cases, there were multiple versions of items reflecting possible different wordings. An additional section was added to the list of items reflecting suggestions made by panellists in Round 1. For items that were to be re-rated, panellists were provided with the median panel rating (and classification), whether there was disagreement within the panel and their own previous rating. As with Round 1, after the completion of each round, the research team reviewed scores and free text comments. Following Round 2 the team discussed which items would be taken forward to Round 3. Following Round 3 the team assessed whether a sufficient consensus existed and made final recommendations to be made based on the Delphi findings.

### Assessment of disagreement

Disagreement about items was based on the inter-percentile range (the difference between the 30th and 70th percentile appropriateness ratings), and the inter-percentile range adjusted for symmetry. The inter-percentile range adjusted for symmetry recognises that when the distribution of ratings is symmetric, the inter-percentile range required to label an indication as disagreement is smaller than when the distribution of ratings is asymmetric. Thus, the inter-percentile range adjusted for symmetry includes a correction factor for asymmetry and is defined as

IPRAS=IPRr+(AI×CFA)

where IPRr is the inter-percentile range required for disagreement when perfect symmetry exists,

AI is the asymmetry index, and CFA is the correction factor for asymmetry. Here AI is calculated as the distance between the central point of the inter-percentile range $(p_{30}+p_{70})/2$ and the central point of the scale (i.e. 5 on a 1–9-point scale). Following Fitch et al.,^10^ we used values of 2.35 and 1.5 for IPRr and CFA, respectively, which were shown to be optimal. We considered there to be disagreement between panellists when the inter-percentile range was more than the inter-percentile range adjusted for symmetry.

### Round 1

#### Definition of clinical ‘excellence’

Panellists were presented with five possible approaches to benchmarking. For one of these – ‘Applicants could be benchmarked against all clinicians working in similar jobs/roles’ – there was a consensus view that it was an appropriate approach providing a favoured way forward. For one other approach – applicants could be benchmarked against all clinicians applying for clinical excellence awards – it was clear that there were problems with this approach related to the potential inclusivity of the process. For example, one respondent noted that this approach would involve ‘comparing with a self-selecting group who may or may not epitomise excellence–e.g. some excellent people may not apply’. Furthermore, there were concerns that the quality of applications may have changed over time. Given these concerns and the distributions of scores for this item, it was not carried forward to Round 2. For one other approach, respondent comments highlighted a specific issue with the wording of the item (which referred to all UK clinicians when the clinical excellence award scheme only applies to England and Wales and not all clinicians are eligible to apply). This item was reworded before being carried forward into Round 2. With the two final approaches (benchmarking against clinicians of similar experience and judging on own merits rather than benchmarking) there was overall uncertainty over the appropriateness, and they were also carried forward into Round 2.

There was consensus that the current definition of clinical excellence used by the Advisory Committee on Clinical Excellence Awards was appropriate and this was not investigated further. However, there was disagreement over whether the definition of excellence should be amended for part-time workers with panellists commenting that this was not appropriate and that ‘There can only be one definition of clinical excellence’*.* A number of panellists made the point that although the definition of excellence should not change, scores did need to reflect the part-time or full-time nature of the applicant’s role. Given this, two further items on the scoring of part-time workers were included going forwards. There was consensus that national nominating bodies, specialist societies and Advisory Committee on Clinical Excellence Awards subcommittees should publish their anonymised data, scoring methodology and justification of their internal processes and this was not considered further.

#### Scoring scale descriptors

There was consensus that it was appropriate to scale descriptors for any future scale in terms of expectations relative to a job description, the reach of an applicant’s contribution to National Health Service care, the significance of an applicant’s contribution to National Health Service care, the impact of an applicant’s contribution to National Health Service care, and that the scale descriptors should be based on these multiple aspects simultaneously. Some panellists made the point that international reach was potentially not the correct aspiration in the context of clinical excellence awards and that national reach might be more important. On this basis, we asked panellists to re-rate the item focused on multiple aspects of excellence, as well as an amended version which did not distinguish between national and international reach at the highest point on the scale. Finally, there was disagreement over an approach to scoring based on statistical definitions. The item was reconsidered in Round 2 with some further guidance on how this might work practically, along with an alternative version taking on panellists’ comments.

#### Scoring

Four approaches to scoring were rated. There was consensus that an evenly spaced large number of possible scores, for example, 0–100, was inappropriate. There was also consensus that an approach aligned to the current system was appropriate with other approaches rated as ‘unsure’. Given that issues have been identified with the current system, and the desire to change we asked panellists to re-rate the three approaches rated ‘unsure’ or ‘appropriate’, with further guidance on the shortcomings of the existing scale. In response to panel member comments, two further items were added considering two different approaches to applying anchor points to a 0 to 10 scale.

There appeared to be a split in opinion over whether the lowest point on the scale should reflect someone performing at, or below, expectations with neither approach being rated appropriate. Panellists were asked to rate these again reflecting on comments made by others. Similarly, there was no consensus on the best way to approach scoring when considering whether all domains should contribute to an applicant’s score, whether the four highest scoring domains should contribute, or whether the applicant should choose to only supply evidence in their top scoring domains and these were returned to the panel for rating again in Round 2. Where there was consensus was that the domains concerning service development and delivery and panellists did not re-rate this item in Round 2. Finally, some panellists suggested alternative scoring approaches based on either an additional global score or a forced ranking approach. Given these suggestions, three further items were included in Round 2 concerning these alternative approaches.

### Round 2

#### Definition of clinical 'excellence’

There were three approaches to benchmarking candidates where there was consensus of acceptability. These were that applicants could be benchmarked against:

- All clinicians working in similar jobs/roles.
- All clinicians, eligible for an award, working in similar jobs/roles.
- All clinicians working in this speciality in similar jobs/roles.

There was also consensus that an approach whereby ‘Applicants should be judged on their own merits and not be benchmarked against their peers’ was not appropriate. There was also a single approach in relation to the definition of excellence and scoring of part-time workers, where consensus was reached which was that:

*The definition of clinical excellence used in the assessment of applications for clinical excellence awards should be the same for all applicants, but that the scoring of applications made by part-time workers should be amended to reflect the part-time nature of their role, with the financial reward made on a pro-rata basis.*

Given a clear acceptable set of approaches was found, the subject of the definition of excellence was not revisited in Round 3.

#### Scoring scale descriptors

A single approach to scale descriptors had a consensus of acceptability, which was that for each domain, the description of scale points could be based on multiple aspects simultaneously, for example, ‘an applicant scoring at the highest point on the scale would be seen to be making an outstanding contribution, which is substantially exceeding the expectation of job description, highly impactful, highly significant, and of national or international reach’. Scoring scales were not re-examined in Round 3.

#### Scoring

Two scoring scale approaches had a consensus of acceptability. One of these was an approach in line with the current scoring system. Given the purpose of the process was to develop a new scale, this was not taken forward to Round 3. There was also a continued lack of consensus as to whether the lowest point on a scale should reflect someone working at, or below, expectations. Given this, we asked panellists to rate two new items based on the acceptable approach (an evenly spaced larger number of possible scores should be allowed to permit more granularity, e.g. 0, 1, 2, 3, 4, 5, 6, 7, 8, 9 or 10 with clearly defined scale descriptors covering a range of points) with scale descriptors assigned reflecting the two different approaches to the lowest point on the scale.

As in Round 2, all three approaches concerning whether four or five domains should count towards an applicant’s final score, and how these domains were chosen, were rated as ‘unsure’. Again, these were returned to the panel in Round 3 along with further comments made by the panel members for reflection. The alternative approaches to scoring based on forced ranking, as suggested by a panel member, were rated as ‘inappropriate’ and were not considered in Round 3. The alternative approach including an additional global score was rated as ‘unsure’ and so panellists were invited to re-rate it in Round 3.

### Round 3

Only six items were considered by the panel in Round 3. Of the two approaches to scoring considered, the version where the lowest point on the scale reflected performance below expectations was rated ‘appropriate’ (median rating 7), while the alternative was rated ‘unsure’ (median rating 6). However, there was some disagreement among panellists for the approach rated ‘appropriate’ with the inter-percentile range (4) slightly higher than the inter-percentile range adjusted for symmetry (3.85). Examination of the histogram (not shown) shows that a small number of panellists considered this approach to be inappropriate. Of the three approaches concerning whether four or five domains should count towards an applicant’s final score, and how these domains were chosen, only one was rated ‘appropriate’ (with the other two rated ‘unsure’), which was that applicants should submit evidence in all five domains, but that just the top four scoring domains should contribute towards their score. Finally, the alternative approach of including a single global score in addition to the individual domain scores was rated as ‘unsure’. Many panellists raised serious concerns over this approach with one commenting ‘I literally cannot think of a more useful tool for the subtly racist, sexist or jobs-for-the-boys scorer to have at their disposal. I think it would be morally, and for all I know, legally indefensible to introduce this system’.

## Appendix B. Further details of the shadow-scoring exercise

### Development of a revised scoring system

Drawing on the results of the Delphi process, a revised scoring system and scale descriptors were developed, which might be applied to each of the five domains proposed under revised scoring arrangements. The scale’s scoring range is between 0 and 10, where 0 is taken to mean that an applicant does not meet some or all of their job plan in the respective domains. The descriptors accompanying the scale draw on four ‘areas of relevance’ identified in earlier phases of the research as being of central importance in assessing the evidence presented by the applicant, namely: performance in relation to expectations of the applicant’s job plan; demonstration of reach of contribution; demonstration of significance of contribution; and overall summary description of contribution.

### Development of training cases

To support the shadow-scoring exercise, we developed a portfolio of training cases to be used in an exercise incorporating a potentially revised approach to scoring.

We first approached the Advisory Committee on Clinical Excellence Awards to test the potential availability of anonymised training cases based on actual historical applications. Initial interactions suggested that this was possible, although we became aware that caution and careful editing were required with respect to the anonymisation of those cases. In addition, our preliminary review of potential cases identified the importance of varying approaches to the inclusion/exclusion of citation material, the inclusion/exclusion of ranking tables reporting the findings of national nominating bodies and specialist societies, and issues relating to the presentation of citations of research material, the latter leading us to identify the importance of the number of authors, and the position of the applicant within the author list and as a lead author (first, corresponding or last author).

Having secured a range of cases we checked this against a requirement that the cases should cover a range of applications that historically proved to be successful or unsuccessful and to cover an appropriate range of new and renewal applications at various levels of award.

Having undertaken this detailed exercise, our final portfolio of training cases for the shadow scoring exercise represented 20 applications. For 18 of these, two versions were created, one including citation material, and a second which had been manipulated to remove citation material. A further two applications without citation material were also anonymised. Thus, a total of 38 training cases were produced.

### Further details of methods

Prior to being sent training cases all participating assessors were required to sign a confidentiality agreement given that it is not possible or desirable to completely anonymise cases. Assessors were sent cases to score in the week commencing 15 November 2021 and were asked to complete the exercise within three weeks. Where the exercise was not completed within that timeframe, assessors were given a further week to complete the exercise. Assessors were paid an honorarium of £500 to compensate them for their time on completion of the exercise.

In addition to scoring the applications, assessors were asked to provide feedback on the guidance revised scoring and descriptors. This included questions on the usefulness of citation and ranking, the ease of application of the scoring template and domain-specific guidance and the estimated time taken to assess each application (Box 3). After the assessors had scored all applications, we asked them to provide any further reflections that they had on using the proposed scoring scale, and associated guidance, in the shadow-scoring exercise.

Box 3. Questions asked of assessors when scoring applications

In assessing the performance of the new scoring scale, parallel analyses have been performed on the scores from both this shadow scoring exercise and from the historic scores received by the applications when originally scored. Multi-level regression models were used to examine the sources of variance in scores given to applications, specifically contributions attributable to differences in the quality of the application itself, contributions due to some assessors scoring systematically higher or lower on all applications, and unexplained or residual variance. The total score given by one assessor for one application was used as the outcome variable in our models (i.e. the sum of scores for all five domains with a maximum value of 50). Random intercepts were included for the application and the assessor. These random effects were crossed rather than nested, reflecting the fact that the same assessor would score more than one application. From these models three sources of variance were identified – the application $\left(\sigma_{\mathrm{AP}}^{2}\right)$ , the assessor $\left(\sigma_{\mathrm{AS}}^{2}\right)$ and residual or error variance $\left(\sigma_{e}^{2}\right)$ . In the context of clinical excellence award scoring, the variance attributed to assessors is of lesser significance. This is because the assessors’ scoring applications which are compared with each other are consistent across applications, and thus subject to the same hawkish and dovish tendencies. For this reason, our prime comparison is in the percentage of variance attributable to either applications or residual that is attributable to the application i.e. $100\times \sigma_{\mathrm{AP}}^{2}/(\sigma_{\mathrm{AP}}^{2}+\sigma_{e}^{2})$ . Confidence intervals on this percentage were estimated using bootstrapping. The higher this percentage attributable to application, the more reliable a scoring system is at distinguishing good and poor performance. To examine whether reliability was dependent on performance (i.e. is it easier to distinguish between two applications scoring highly than two applications scoring low) we also estimated the correlation between the variance in scores of different assessors for the same application and the mean of the scores given by different assessors to the same application.

## Appendix C. Assessors’ feedback on the use of the new scoring scheme and accompanying guidance

Table A1 shows the responses to the statements presented to the assessors when scoring applications (responses of not applicable have been excluded). In most occurrences, the assessors felt the scoring template and domain-specific guidance were easy to apply (83% and 84% agreed or strongly agreed, respectively). They also felt that most of the time (70% agreed or strongly agreed) the job plan provided a clear understanding of the applicant's roles. Similarly, most of the time it was felt that citations were useful (66% agreed or strongly agreed) with somewhat less agreeing that ranking of citation material was useful (52% agreed or strongly agreed). The most common time spent coming to an assessment of an application was 10–20 min (45% of assessments) with 11% taking less than 10 min, 13% taking 40–60 min and 2% taking over an hour.

**Table A1.** Assessor ratings (*n*[%]) of agreements with statements referring to the application of the new scoring system.

	Strongly disagree	Disagree	Neither agree nor disagree	Agree	Strongly agree
The job plan provided a clear understanding of applicant's roles and expected contribution	25 (5.3%)	61 (13.0%)	53 (11.3%)	222 (47.1%)	110 (23.4%)
Any provided citation material was useful in informing my scoring*	15 (6.8%)	12 (5.5%)	48 (21.8%)	91 (41.4%)	54 (24.6%)
Any provided ranking of citation material was useful in informing my scoring*	6 (4.3%)	8 (5.7%)	53 (37.6%)	43 (30.5%)	31 (22.0%)
The scoring template provided in the guide to assessors was easy to apply to this application	3 (0.7%)	23 (5.0%)	54 (11.8%)	259 (56.4%)	120 (26.1%)
The domain-specific guidance provided in the guide to assessors was easy to apply to this application	1 (0.2%)	17 (3.7%)	57 (12.3%)	276 (59.5%)	113 (24.4%)

*Restricted to cases where citation material was present.

A range of free text comments were submitted based on assessors’ experiences of using the revised scoring scheme. Looking across these for consistent or diverging views, three main points could be drawn:

1. Applicants vary in how detailed they write their job plans, assessors reflected that when written very well, the assessment process is smooth, whereas when they’re vague (e.g. not reporting how many supporting professional activity sessions are involved and remunerated, how much of the applicants’ time was attributed to direct clinical care, or simply listing all of their roles but not spelling out what the roles involve day-to-day), it is much more challenging. Clearer guidance and support for applicants on writing a detailed job plan/what to involve may help this.
2. Views on the value of citations were mixed. Some are described as helpful, especially when the application isn’t written very well, as it provides more info/context, whereas others say they don't really take notice of them/they're always positive. Citations from reliable sources such as Chief Executive Officers, Colleges and funding bodies were described as most helpful by some assessors. Clear guidance for assessors on how much value to place on citations may be helpful, as well as instructions for applicants on what type of citations/from whom should be sought.
3. Assessors reflected that using the new scoring template meant taking more time than usual because of the need to become familiar with the new scores and scale descriptors. Clear guidance and a recommendation to allow for more time when using a new scale may be helpful.
