# Supplementary material for: Informing the development of a scoring system for National Health Service Clinical Impact Awards; a Delphi process and simulated scoring exercise
Source: JRSM Open. 2024 Jan 14;15(1):20542704231217887. doi: 10.1177/20542704231217887 (PMC10790597; doi:10.1177/20542704231217887)
Supplement: sj-docx-1-shr-10.1177_20542704231217887 - Supplemental material for Informing the development of a scoring system for National Health Service Clinical Impact Awards; a Delphi process and simulated scoring exercise [file sj-docx-1-shr-10.1177_20542704231217887.docx]

# Appendix A – Further details of the Delphi process

## Methods

In Round 1 (15 July 2021 – 29 July 2021) panellists were provided with a summary of academic literature pertaining to Clinical Excellence Awards, the findings of the INCEA project evidence review into performance-related financial incentive schemes, and a summary of the preliminary findings from the qualitative interviews conducted at that time. They were then asked to rate and comment on 25 items pertaining to the definition of excellence and potential scoring approaches. These items were based on discussions between the research team and informed by qualitative work. After completion of the round the research team reviewed scores and free text comments and discussed which items would be taken forward to subsequent rounds.

In Round 2 (19 August 2021 – 2 September 2021) and Round 3 (23 September 2021 – 11 October 2021) panellists were provided with a written summary of the findings from the previous round. This included both a summary of the appropriate ratings of items, and a summary of the free text comments including a selection of illustrative quotes. Items were not presented for rating again when the median was in either ‘appropriate’, or ‘inappropriate’ and there was no disagreement on the item and no comments that researchers judged required review by the panel. Some item wordings were altered following suggestions by panellists, and in some cases, there were multiple versions of items reflecting possible different wordings. An additional section was added to the list of items reflecting suggestions made by panellists in Round 1. For items that were to be rerated, panellists were provided with the median panel rating (and classification), whether there was disagreement within the panel, and their own previous rating. As with round 1, after completion of each round the research team reviewed scores and free text comments. Following Round 2 the team discussed which items would be taken forward to Round 3. Following Round 3 the team assessed whether a sufficient consensus existed and made final recommendations to be made based on the Delphi findings.

## Assessment of disagreement

Disagreement about items was based on the inter-percentile range (IPR) (the difference between the 30th and 70th percentile appropriateness ratings), and the inter-percentile range adjusted for symmetry (IPRAS). The IPRAS recognises that when the distribution of ratings are symmetric, the IPR required to label an indication as disagreement is smaller than when the distribution of ratings are asymmetric. Thus, the IPRAS includes a correction factor for asymmetry and is defined as

$$IPRAS=IPRr+\left( AI\times CFA \right)$$

Where IPRr is the inter-percentile range required for disagreement when perfect symmetry exists,

AI is the asymmetry index, and CFA is the correction factor for asymmetry. Here AI is calculated as the distance between the central point of the IPR $\left( \frac{p_{30}+p_{70}}{2} \right)$and the central point of the scale (i.e.

5 on a 1–9-point scale). Following Fitch et al. we used values of 2.35 and 1.5 for IPRr and CFA respectively which were shown to be optimal.^1^ We considered there to be disagreement between panellists when the IPR was more than the IPRAS.

## Round 1

##### Definition of clinical ‘excellence’

Panellists were presented with five possible approaches to benchmarking. For one of these – “Applicants could be benchmarked against all clinicians working in similar jobs/roles” – there was a consensus view that it was an appropriate approach providing a favoured way forward. For one other approach – applicants could be benchmarked against all clinicians applying for clinical excellence awards – it was clear that there were problems with this approach related to the potential inclusivity of the process. For example, one respondent noted that this approach would involve “*comparing with a self-selecting group who may or may not epitomise excellence – e.g. some excellent people may not apply*”. Furthermore, there were concerns that the quality of applications may have changed over time. Given these concerns and the distributions of scores for this item, it was not carried forwards to Round 2. For one other approach, respondent comments highlighted a specific issue with the wording of the item (which referred to all UK clinicians when the clinical excellence award scheme only applies to England and Wales and not all clinicians are eligible to apply). This item was reworded before being carried forward into Round 2. With the two final approaches (benchmarking against clinicians of similar experience and judging on own merits rather than benchmarking) there was overall uncertainty over the appropriateness, and they were also carried forward into Round 2.

There was consensus that the current definition of clinical excellence used by ACCEA was appropriate and this was not investigated further. However, there was disagreement over whether the definition of excellence should be amended for part-time workers with panellists commenting that this was not appropriate and that “*There can only be one definition of clinical excellence.*” A number of panellists made the point that although the definition of excellence should not change, scores did need to reflect the part-time or full-time nature of the applicant’s role. Given this, two further items on the scoring of part-time workers. There was consensus that national nominating bodies, specialist societies and ACCEA subcommittees should publish their anonymised data, scoring methodology and justification of their internal processes and this was not considered further.

##### Scoring scale descriptors

There was consensus that it was appropriate to scale descriptors for any future scale in terms of expectations relative to job description, the reach of an applicant’s contribution to NHS care, the significance of an applicant’s contribution to NHS care, the impact of an applicant’s contribution to NHS care, and that the scale descriptors should be based on these multiple aspects simultaneously. Some panellists made the point that international reach was potentially not the correct aspiration in the context of clinical excellence awards and that national reach might be more important. On this basis we asked panellists to re-rate the item focussed on multiple aspects of excellence, as well as an amended version which did not distinguish between national and international reach at the highest point on the scale. Finally, there was disagreement over an approach to scoring based on statistical definitions. The item was reconsidered in Round 2 with some further guidance on how this might work practically, along with an alternative version taking on panellists’ comments.

##### Scoring

Four approaches to scoring were rated. There was consensus that an evenly spaced large number of possible scores, e.g. 0-100, was inappropriate. There was also consensus that an approach aligned to the current system was appropriate with other approaches rated as ‘unsure’. Given that issues have been identified with the current system, and the desire to change we asked panellists to re-rate the three approaches rated ‘unsure’ or ‘appropriate’, with further guidance on the short comings of the existing scale. In response to panel member comments two further items were added considering two different approaches to applying anchor points to a 0 to 10 scale.

There appeared to be a split in opinion over whether the lowest point on the scale should reflect someone performing at, or below, expectations with neither approach being rated appropriate. Panellists were asked to rate these again reflecting on comments made by others. Similarly, there was no consensus on the best way to approach scoring when considering whether all domains should contribute to an applicant’s score, whether the four highest scoring domains should contribute, or whether the applicant should choose to only supply evidence in their top scoring domains and these were returned to the panel for rating again in Round 2. Where there was consensus was that the domains concerning service development and delivery and panellists did not re-rate this item in Round 2. Finally, some panellists suggested alternative scoring approaches based on either an additional global score or a forced ranking approach. Given these suggestions three further items were included in Round 2 concerning these alternative approaches.

## Round 2

##### Definition of clinical 'excellence’

There were three approaches to benchmarking candidates where there was consensus of acceptability. These were that applicants could be benchmarked against:

- all clinicians working in similar jobs/roles
- all clinicians, eligible for an award, working in similar jobs/roles
- all clinicians working in this specialty in similar jobs/roles

There was also consensus that an approach whereby “Applicants should be judged on their own merits and not be benchmarked against their peers” was not appropriate. There was also a single approach in relation to the definition of excellence and scoring of part-time workers, where consensus was reached which was that:

*The definition of clinical excellence used in the assessment of applications for clinical excellence awards should be the same for all applicants, but that the scoring of applications made by part-time workers should be amended to reflect the part-time nature of their role, with the financial reward made on a pro-rata basis*.

Given a clear acceptable set of approaches was found the subject of the definition of excellence was not revisited in Round 3.

##### Scoring scale descriptors

A single approach to scale descriptors had consensus of acceptability, which was that, for each domain the description of scale points could be based on multiple aspects simultaneously, e.g., ‘an applicant scoring at the highest point on the scale would be seen to be making an outstanding contribution, which is substantially exceeding the expectation of job description, highly impactful, highly significant, and of national or international reach’. Scoring scales were not re-examined in Round 3.

##### Scoring

Two scoring scale approaches had consensus of acceptability. One of these was an approach in line with the current scoring system. Given the purpose of the process was to develop a new scale, this was not taken forward to Round 3. There was also a continued lack of consensus as to whether the lowest point on a scale should reflect someone working at, or below, expectations. Given this we asked panellists to rate two new items based on the acceptable approach (an evenly spaced larger number of possible scores should be allowed to permit more granularity, e.g., 0, 1, 2, 3, 4, 5, 6, 7, 8, 9 or 10 with clearly defined scale descriptors covering a range of points) with scale descriptors assigned reflecting the two different approaches to the lowest point on the scale.

As in Round 2, all three approaches concerning whether four or five domains should count towards an applicant’s final score, and how these domains were chosen, were rated as ‘unsure’. Again, these were returned to the panel in Round 3 along with further comments made by panel-members for reflection. The alternative approaches to scoring based on forced ranking, as suggested by a panel member, were rated as ‘inappropriate’ and were not considered in Round 3. The alternative approach including an additional global score was rated as ‘unsure’ and so panellists were invited to re-rate it in Round 3.

## Round 3

Only six items were considered by the panel in Round 3. Of the two approaches to scoring considered, the version where the lowest point on the scale reflected performance below expectations was rated ‘appropriate’ (median rating 7), whilst the alternative was rated ‘unsure’ (median rating 6). However, there was some disagreement among panellists for the approach rated ‘appropriate’ with the IPR (4) slightly higher than the IPRAS (3.85). Examination of the histogram (not shown) shows that a small number of panellists considered this approach to be inappropriate. Of the three approaches concerning whether four or five domains should count towards an applicant’s final score, and how these domains were chosen, only one was rated ‘appropriate’ (with the other two rated ‘unsure’) which was that applicants should submit evidence in all five domains, but that just the top four scoring domains should contribute towards their score. Finally, the alternative approach of including a single global score in addition to the individual domains scores was rated as ‘unsure’. Many panellists raised serious concerns over this approach with one commenting “*I literally cannot think of a more useful tool for the subtly racist, sexist or jobs-for-the-boys scorer to have at their disposal. I think it would be morally, and for all I know, legally indefensible to introduce this system*.”

# Appendix B – Further details of the shadow scoring exercise

### Development of a revised scoring system

Drawing on the results of the Delphi process a revised scoring system and scale descriptors was developed which might be applied to each of the five domains proposed under revised scoring arrangements. The scale’s scoring range is between 0 and 10, where zero is taken to mean that an applicant does not meet some or all of their job plan in the respective domains. The descriptors accompanying the scale draw on four ‘areas of relevance’ identified in earlier phases of the research as being of central importance in assessing evidence presented by the applicant, namely: performance in relation to expectations of the applicant’s job plan; demonstration of reach of contribution; demonstration of significance of contribution; and overall summary description of contribution.

### Development of training cases

To support the shadow scoring exercise, we developed a portfolio of training cases to be used in an exercise incorporating a potentially revised approach to scoring.

We first approached ACCEA to test the potential availability of anonymised training cases based on actual historical applications. Initial interactions suggested that this was possible, although we became aware that caution and careful editing was required in respect on anonymisation of those cases. In addition, our preliminary review of potential cases identified the importance of varying approaches to the inclusion/exclusion of citation material, the  inclusion/exclusion of ranking tables reporting the findings of national nominating bodies and specialist societies, and issues relating to the presentation of citations of research material, the latter leading us to identify the importance of the number of authors, and the position of the applicant within the author list and as a lead author (first, corresponding or last author).

Having secured a range of cases we checked this against a requirement that the cases should cover a range of applications that historically proved to be successful or unsuccessful, and to cover an appropriate range of new and renewal applications at various levels of award.

Having undertaken this detailed exercise, our final portfolio of training cases for the shadow scoring exercise represented 20 applications. For 18 of these, two versions were created, one including citation material, and a second which had been manipulated to remove citation material. A further two applications without citation material were also anonymised. Thus, a total of 38 training cases were produced.

### Further detail of methods

Prior to being sent training cases all participating assessors were required to sign a confidentiality agreement given that it is not possible or desirable to completely anonymise cases. Assessors were sent cases to score in the week commencing 15th November 2021 and were asked to complete the exercise within three-weeks. Where the exercise was not completed within that timeframe, assessors were given a further week to complete the exercise. Assessors were paid an honorarium of £500 to compensate them for their time on completion of the exercise.

In addition to scoring the applications, assessors were asked to provide feedback on the guidance revised scoring and descriptors. This included questions on the usefulness of citation and ranking, the ease of application of the scoring template and domain specific guidance and the estimated time taken to assess each application (Box). After the assessors had scored all applications, we asked them to provide any further reflections that they had on using the proposed scoring scale, and associated guidance, in the shadow scoring exercise.

*Box Questions asked of assessors when scoring applications*

- Please rate to what extent you agree with the following statements for this particular application. (Response options: Strongly disagree, Disagree, Neither agree nor disagree, Agree, Strongly agree, Not applicable)
  - The job plan provided a clear understanding of applicant's roles and expected contribution
  - Any provided citation material was useful in informing my scoring
  - Any provided ranking of citation material was useful in informing my scoring
  - The scoring template provided in the guide to assessors was easy to apply to this application
  - The domain specific guidance provided in the guide to assessors was easy to apply to this application
- Please also provide us with any comments you have on the aspects in question.
- Approximately how long did you spend in coming to an assessment of this application? (Response options; Less than 5 minutes, 5 to 10 minutes, 10 to 20 minutes, 20 to 40 minutes, 40 to 60 minutes, Over 1 hour)
- In your view, how likely is this application to be successful? i.e. to achieve a new award, or achieve renewal of an existing award. (Response options; Very unlikely to be successful, Unlikely to be successful, Likely to be successful, Very likely to be successful).
- Do you have any further comments specific to scoring this application?

In assessing the performance of the new scoring scale, parallel analyses have been performed on the scores from both this shadow scoring exercise and from the historic scores received by the applications when originally scored. Multi-level regression models were used to examine the sources of variance in scores given to applications, specifically contributions attributable to differences in the quality of the application itself, contributions due to some assessors scoring systematically higher or lower on all applications, and unexplained or residual variance. The total score given by one assessor for one application was used as the outcome variable in our models (i.e. the sum of scores for all five domains with a maximum value of 50). Random intercepts were included for application and for assessor. These random effects were crossed rather than nested, reflecting the fact that the same assessor would score more than one application. From these models three sources of variance were identified– the application ($\sigma_{AP}^{2})$, the assessor ($\sigma_{AS}^{2})$ and residual or error variance ($\sigma_{e}^{2})$. In the context of clinical excellence award scoring the variance attributed to assessors is of lesser significance. This is because the assessors’ scoring applications which are compared to each other are consistent across applications, and thus subject to the same hawkish and dovish tendencies. For this reason, our prime comparison is in the percentage of variance attributable to either applications or residual that is attributable to the application i.e. $100\times\sigma_{AP}^{2}/(\sigma_{AP}^{2}+\sigma_{e}^{2})$. Confidence intervals on this percentage were estimated using bootstrapping. The higher this percentage attributable to application, the more reliable a scoring system is at distinguishing good and poor performance. To examine whether reliability was dependant on performance (i.e. is it easier to distinguish between two applications scoring highly than two applications scoring low) we also estimated the correlation between the variance in scores of different assessors for the same application and the mean of the scores given by different assessors to the same application.

# Appendix C – Assessors’ feedback on use of new scoring scheme and accompanying guidance

Table A1 shows the responses to the statements presented to the assessors when scoring applications (responses of not applicable have been excluded). In most occurrences the assessors felt the scoring template and domain specific guidance were easy to apply (83% and 84% agreed or strongly agreed respectively). They also felt that most of the time (70% agreed or strongly agreed) the job plan provided a clear understanding of the applicant's roles. Similarly, most of the time it was felt that citations were useful (66% agreed or strongly agreed) with somewhat less agreeing that ranking of citation material was useful (52% agreed or strongly agreed). The most common time spent coming to an assessment of an application was 10-20 minutes (45% of assessments) with 11% taking less than 10 minutes, 13% taking 40-60 minutes and 2% taking over an hour.

Table A1: Assessor ratings (n(%)) of agreements with statements referring to the application of the new scoring system.

|  | Strongly disagree | Disagree | Neither agree nor disagree | Agree | Strongly agree |
| --- | --- | --- | --- | --- | --- |
| The job plan provided a clear understanding of applicant's roles and expected contribution | 25  (5.3%) | 61 (13.0%) | 53 (11.3%) | 222 (47.1%) | 110 (23.4%) |
| Any provided citation material was useful in informing my scoring* | 15  (6.8%) | 12  (5.5%) | 48 (21.8%) | 91 (41.4%) | 54 (24.6%) |
| Any provided ranking of citation material was useful in informing my scoring* | 6  (4.3%) | 8  (5.7%) | 53 (37.6%) | 43 (30.5%) | 31 (22.0%) |
| The scoring template provided in the guide to assessors was easy to apply to this application | 3  (0.7%) | 23  (5.0%) | 54 (11.8%) | 259 (56.4%) | 120 (26.1%) |
| The domain specific guidance provided in the guide to assessors was easy to apply to this application | 1  (0.2%) | 17  (3.7%) | 57 (12.3%) | 276 (59.5%) | 113 (24.4%) |
| *Restricted to cases where citation material was present | | | | | |
|  | | | | | |

A range of free text comments were submitted based on assessors’ experiences of using the revised scoring scheme. Looking across these for consistent or diverging views, three main points could be drawn:

1. Applicants vary in how detailed they write their job plans, assessors reflected that when written very well, the assessment process is smooth, whereas when they're vague (e.g. not reporting how many supporting professional activity sessions are involved and remunerated, how much of the applicants’ time was attributed to direct clinical care, or simply listing all of their roles but not spelling out what the roles involve day-to-day), it is much more challenging. Clearer guidance and support for applicants on writing a detailed job plan/what to involve, may help this.
2. Views on the value of citations were mixed. Some described as helpful, especially when the application isn't written very well, as it provides more info/context, whereas others say they don't really take notice of them/they're always positive. Citations from reliable sources such as CEOs, Colleges and funding bodies were described as most helpful by some assessors. Clearer guidance for assessors on how much value to place on citations may be helpful, as well as instructions for applicants on what type of citations/from who should be sought.
3. Assessors reflected that using the new scoring template meant taking more time than usual because of the need to become familiar with the new scores and scale descriptors. Clear guidance and a recommendation to allow for more time when using a new scale may be helpful.

## Reference

1. Fitch K, Bernstein S, Aguilar M, et al. The RAND/UCLA Appropriateness Method User's Manual. 2001.
